# Study protocol for a Randomised controlled trial of EArly transjugular intrahepatiC porTosystemic stent–shunt in Acute Variceal Bleeding (REACT-AVB trial)

**DOI:** 10.1136/bmjgast-2023-001314

**Published:** 2024-03-22

**Authors:** Dhiraj Tripathi, David Patch, Homoyon Mehrzad, Dominic Yu, Richard J Aspinall, Matthew J Armstrong, Adrian Stanley, Hamish Ireland, Simon Travis, Peter Hayes, Mandy Lomax, Nicholas Roslund, Emily Lam, Gemma Slinn, Sue Jowett, Catherine Moakes, Alisha Maher, Elizabeth Brettell, Sukhwant Sehmi

**Affiliations:** 1 Liver Unit, University Hospitals Birmingham NHS Foundation Trust, Birmingham, Birmingham, UK; 2 Institute of Immunology and Immunotherapy, University of Birmingham, Birmingham, UK; 3 Hepatology and Liver Transplantation, Royal Free Hampstead NHS Trust, London, UK; 4 Interventional Radiology, University Hospitals Birmingham NHS Foundation Trust, Birmingham, Birmingham, UK; 5 Department of Radiology, Royal Free London NHS Foundation Trust, London, UK; 6 Gastroenterology and Hepatology, Portsmouth Hospitals NHS Trust, Portsmouth, UK; 7 GI Unit, Glasgow Royal Infirmary, Glasgow, Glasgow, UK; 8 Department of Radiology, Royal Infirmary of Edinburgh, Edinburgh, UK; 9 Department of Radiology, Nottingham University Hospitals NHS Trust, Nottingham, UK; 10 Hepatology, Royal Infirmary of Edinburgh, Edinburgh, UK; 11 Walton-on-Thames, Walton-on-Thames, UK; 12 London, London, UK; 13 GI and Liver PPI Group, University Hospitals Birmingham NHS Foundation Trust, Birmingham, Birmingham, UK; 14 Birmingham Clinical Trials Unit, University of Birmingham, Birmingham, West Midlands, UK; 15 Institute of Applied Health Research, University of Birmingham, Birmingham, West Midlands, UK

**Keywords:** CIRRHOSIS, PORTAL HYPERTENSION, OESOPHAGEAL VARICES, GASTROINTESTINAL BLEEDING, STENTS

## Abstract

**Introduction:**

In liver cirrhosis, acute variceal bleeding (AVB) is associated with a 1-year mortality rate of up to 40%. Data on early or pre-emptive transjugular intrahepatic portosystemic stent–shunt (TIPSS) in AVB is inconclusive and may not reflect current management strategies. Randomised controlled trial of EArly transjugular intrahepatiC porTosystemic stent–shunt in AVB (REACT-AVB) aims to investigate the clinical and cost-effectiveness of early TIPSS in patients with cirrhosis and AVB after initial bleeding control.

**Methods and analysis:**

REACT-AVB is a multicentre, randomised controlled, open-label, superiority, two-arm, parallel-group trial with an internal pilot. The two interventions allocated randomly 1:1 are early TIPSS within 4 days of diagnostic endoscopy or secondary prophylaxis with endoscopic therapy in combination with non-selective beta blockers. Patients aged ≥18 years with cirrhosis and Child-Pugh Score 7–13 presenting with AVB with endoscopic haemostasis are eligible for inclusion. The primary outcome is transplant-free survival at 1 year post randomisation. Secondary endpoints include transplant-free survival at 6 weeks, rebleeding, serious adverse events, other complications of cirrhosis, Child-Pugh and Model For End-Stage Liver Disease (MELD) scores at 6 and 12 months, health-related quality of life, use of healthcare resources, cost-effectiveness and use of cross-over therapies. The sample size is 294 patients over a 4-year recruitment period, across 30 hospitals in the UK.

**Ethics and dissemination:**

Research ethics committee of National Health Service has approved REACT-AVB (reference number: 23/WM/0085). The results will be submitted for publication in a peer-reviewed journal. A lay summary will also be emailed or posted to participants before publication.

**Trial registration number:**

ISRCTN85274829; protocol version 3.0, 1 July 2023.

**WHAT IS ALREADY KNOWN ON THIS TOPIC**

- In patients with cirrhosis, an early transjugular intrahepatic portosystemic stent–shunt (TIPSS) in acute variceal bleeding can result in improved patient survival compared with standard of care. However, the current trials have small sample sizes, and some may not reflect the current standard of care. Moreover, the data on patient selection of high-risk groups is inadequate. An evaluation of early TIPSS with enough patients to determine the effect on transplant-free survival is a priority.

**WHAT THIS STUDY ADDS**

- This study is the largest randomised controlled trial comparing early TIPSS with the current standard of care. The combination of a large sample size and a control arm reflective of current standard of care is a significant strength. In addition, quality of life and cost-effectiveness will be studied.

**HOW THIS STUDY MIGHT AFFECT RESEARCH, PRACTICE OR POLICY**

- If the study showed that early TIPSS resulted in improved patient survival, there would be significant implications for patient care and healthcare systems. Improved patient outcomes could reduce healthcare resource utilisation such as recurrent admissions or the need for liver transplantation. In some healthcare systems, a significant reorganisation may be necessary to accommodate the additional early TIPSS procedures.

## Introduction

### Existing research and current practice

Liver disease is the fifth largest cause of death in the UK. There had been a five-fold increase in the development of cirrhosis in 35–55 year olds between 2003 and 2013, with 30 000–60 000 at risk or affected by liver cirrhosis. Approximately 50% of patients with cirrhosis develop varices, which equates to over 25 000 prevalent cases.^1^ Variceal bleeding has an inpatient mortality rate of 15% and 1-year mortality rate of up to 40%.^2^ Increased hospitalisation results in increased use of secondary care and substantial healthcare costs. Therefore, optimising the management of acute variceal bleeding (AVB) to minimise the risk of variceal rebleeding and improve survival are important clinical and economic goals.

Current clinical practice guidelines recommend endoscopic therapy with variceal band ligation (VBL) for treating acute oesophageal variceal bleeding or tissue adhesives or thrombin injection for acute gastric variceal bleeding in combination with drug therapy (terlipressin and antibiotics).^3 4^ Transjugular intrahepatic portosystemic stent–shunt (TIPSS) is recommended where these measures fail to control bleeding. This well-established emergency strategy is often referred to as ‘rescue’ or ‘salvage’ TIPSS therapy. However, although rescue TIPSS therapy controls variceal bleeding in over 90% of patients, the mortality can be as high as 36% at 6 weeks and 42% at 1 year.^5 6^ Patients with advanced cirrhosis and decompensation (Child-Pugh Score>13, lactate≥12 mmol/L and/or MELD Score≥30) have high levels of mortality and TIPSS in this patient group is likely to be futile.^5 7^

There has been much interest in TIPSS, not as a *rescue* therapy due to ongoing AVB but as an *early (ie, prophylactic or pre-emptive*) therapy in selected high-risk patients after initial control of bleeding with standard endoscopic and drug therapy. The risk of variceal rebleeding can be as high as 60%, with each variceal rebleeding episode associated with 20% mortality, particularly in the first 5 days following a bleed.^8^ Therefore, early interventions during the AVB episode are likely to have the greatest impact on outcomes.

There have been three Randomised controlled trial (RCTs) of early TIPSS in AVB using covered stents. Garcia-Pagan *et al* reported better 12-month transplant-free survival by insertion of early polytetrafluoroethylene covered TIPSS within 72 hours of an index bleed in high-risk patients with predominantly alcohol-related liver disease defined as Childs C or Childs B cirrhosis with active bleeding at endoscopy (n=63, 86% vs 61%, absolute risk reduction 25%, 95% CI: 2% to 48%).^9^ The standard of care was banding in combination with drug therapy. A single-centre RCT from China of early TIPSS (n=86, with TIPSS placed within 72 hours of index endoscopy) versus standard of care (n=46) in patients with Child-Pugh B and C reported better overall transplant-free survival (HR 0·50, 95% CI: 0·25 to 0·98) and improved control of bleeding or rebleeding with early TIPSS (HR 0·26, 95% CI: 0·12 to 0·55).^10^ The benefit was seen in all groups regardless of active bleeding. There was no difference in the incidence of hepatic encephalopathy. It is important to note that in this study, 75% of patients had chronic hepatitis B virus (HBV) infection (33% HBV-DNA negative). Antiviral therapy could have influenced outcomes in addition to TIPSS, and the results are difficult to extrapolate to western populations where alcohol-related liver disease predominates. Furthermore, endoscopic sclerotherapy was used in over 5% of patients, which is not in keeping with current international guidelines which recommend VBL.^3 11^ A further RCT of early TIPSS from the UK in 58 patients with Child-Pugh Score≥8, published in 2020, showed neither difference in 1-year survival (75.9% vs 79.3% for TIPSS arm and control arm, respectively (p=0.79)) nor rebleeding, regardless of severity of liver disease or active bleeding. It is noteworthy that the 1-year transplant-free survival in the standard of care group was significantly better than in the 2010 European study (76% vs 61%).^9^ This would suggest improved overall care of patients with AVB in the last decade, therefore caution is required when extrapolating the results from the European study to the present time. Furthermore, carvedilol was used in most patients in the UK study standard of care group, compared with propranolol in the European study. Carvedilol has a greater effect on portal pressure^12^ and could be a contributing factor to the better outcomes in the UK standard of care group, although further study of carvedilol in secondary prevention of variceal bleeding is required.

The trial by Dunne *et al* highlights the challenges in adherence to the 72-hour window for early TIPSS, with 23/29 patients (79%) actually having a TIPSS and only 13/23 receiving TIPSS within 72 hours, but all within 5 days. It is, however, worth noting that a previous UK RCT of TIPSS versus banding for secondary prevention of variceal rebleeding,^13^ where TIPSS was placed within 72 hours of acute bleeding in all patients, had similar results to the trial by Dunne *et al.* 14 However, the standard of care was not in keeping with current practice and TIPSS was done using bare stents. An important finding of all these RCTs is that rescue TIPSS was necessary in between 10% and 31% in the standard of care groups due to refractory rebleeding and was invariably associated with very poor outcomes.

Data from observational studies has fuelled the debate regarding patient selection for early TIPSS.^15–19^ While patients with Child-Pugh C disease appear to have improved survival following early TIPSS, this is not always the case for Child-Pugh B patients with active bleeding.^15–19^ An observational study also suggested that patients with a MELD Score of ≥19 are likely to benefit from early TIPSS,^17^ a finding confirmed by Lv *et al*.^18^ It is not clear from these studies if there is a maximum threshold of severity of liver disease beyond which there is no benefit from early TIPSS.

A meta-analysis of two RCTs and two observational studies demonstrated that early TIPSS is associated with reduced overall mortality compared with standard of care (OR 0.38, 95% CI: 0.17 to 0.83, p=0.02).^9 15 20–22^ The reduced mortality was only observed in patients with Child-Pugh C Score<14. There was significantly less rebleeding with early TIPSS without significant difference in hepatic encephalopathy. An individual patient data meta-analysis of 7 studies (3 RCTs and 4 observational studies) of 1327 patients showed 6-week and 1-year survival rates were significantly higher in the early TIPSS group than the standard of care group (93% vs 76.8% and 79% vs 62%, respectively, log-rank p<0.001).^10 18–23^ The improved outcomes of early TIPSS were seen in Child-Pugh B and C patients with active bleeding. Number of patients needed to treat to save one life was 4.23 (95% CI: 3.57 to 6.94). There were no significant differences with respect to hepatic encephalopathy. However, both randomised controlled and observational studies were included, and the authors concluded that further prospective studies were necessary. The same group updated this individual patient data meta-analysis,^24^ incorporating the UK RCT,^14^ although a multicentre French audit was not included.^16^ The results confirmed benefit of early TIPSS with improved survival of a similar magnitude in Child-Pugh Score 8–13 patients and in those with Child-Pugh B disease and active bleeding. Another meta-analysis included all four key RCTs,^10 14 20 21^ although it was not based on individual patient data.^25^ Analysis of these RCTs demonstrated no significant difference in mortality at 6 weeks (relative risk (RR) 0.33, 95% CI: 0.08 to 1.36) or at 1 year (RR 0.76, 95% CI: 0.51 to 1.14). The authors recommended a sufficiently powered RCT to answer this question.

### Trial rationale

The research question is ‘Does early TIPSS within 4 days of an acute variceal bleed result in improved transplant-free patient survival when compared with standard of care?’ The data to support universal adoption of early TIPSS in all high-risk groups is currently inadequate. An evaluation of early TIPSS with a sufficient number of patients to determine the effect on transplant-free survival is a priority. In addition, quality of life and cost-effectiveness have not been well studied in previous trials but are important outcomes to assess.

Current clinical practice guidelines produced by the British Society of Gastroenterology (BSG) recommends further research into early TIPSS with particular focus on patient selection. A document entitled ‘NHS England’s Research Needs Assessment 2018’ produced jointly by National Institute of Health and Care Research (NIHR) and National Health Service (NHS) England identified TIPSS and variceal haemorrhage as an area where further research is required.^26^

### Primary objective

The primary clinical objective is to investigate whether early TIPSS improves transplant-free survival at 1 year compared with standard endoscopic plus pharmacological therapy in patients with cirrhosis and AVB after initial control of bleeding by VBL.

### Secondary objectives

These include the effect of early TIPSS compared with standard of care on rebleeding, complications of cirrhosis, progression of liver disease (MELD and Child-Pugh scores), health-related quality of life, use of healthcare resources and safety.

## Methods and analysis

### Randomised controlled trial of EArly transjugular intrahepatiC porTosystemic stent–shunt in AVB (REACT-AVB) trial design

REACT-AVB is a pragmatic, multicentre, randomised controlled, open-label, two-arm, superiority, parallel group trial with an internal pilot. At least 30 acute NHS trusts/health boards in the UK offering 24-hour service for patients with AVB will be involved in trial recruitment. The detailed trial design is described below.

### Eligibility criteria

**Figure 1:**
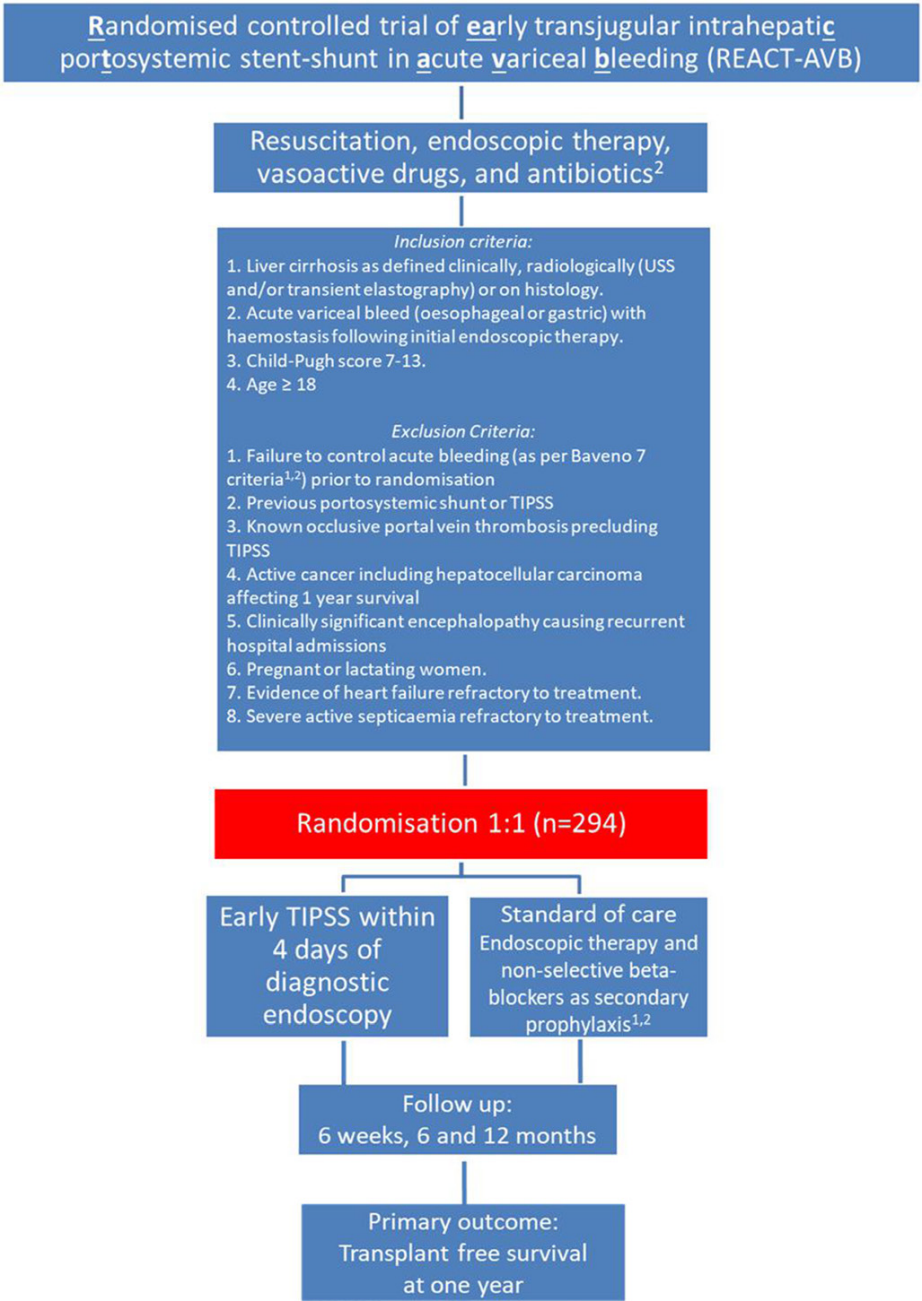
Trial schema.

To be eligible for REACT-AVB, a patient must be aged ≥18 years, have cirrhosis and present with an AVB with endoscopic haemostasis. Patients with failure to control bleeding as defined by Baveno VII criteria^4^ and those with occlusive portal vein thrombosis precluding TIPSS will be excluded. We did not include an upper age limit as there is no clear scientific basis for such an exclusion, for example, absolute age cut-off as a contraindication for TIPSS.^27^ Moreover, our patient and public representatives are in favour of age criteria being inclusive of all adults. Detailed inclusion and exclusion criteria are illustrated in figure 1.

### Internal pilot

An internal pilot phase of 12 months will be undertaken. The aims of the pilot phase will be to assess whether the ‘hub’ and ‘spoke’ model, recruitment rate, randomisation, delivery of the intervention within the specified time and the follow-up assessment schedule are feasible. The objectives for the pilot will be as follows:

- 20 sites (combination of ‘hub’ and ‘spoke’) to be opened during the pilot stage.
- 33 participants to be recruited across the sites.
- >90% of the participants to receive early TIPSS within 4 calendar days of diagnostic endoscopy (intervention arm only).

### Recruitment: hub and spoke model

Regional TIPSS centres will be considered ‘hub’ sites and will be able to randomise participants and perform either treatment intervention. Sites that do not routinely perform TIPSS but routinely refer (as per standard clinical practice) patients for TIPSS procedures will be referred to as ‘spoke’ sites. Spoke sites will be able to randomise participants and perform the standard of care intervention. Hubs will also be expected to accept participants randomised from ‘spoke’ sites who have been allocated to the TIPSS intervention. The spoke will need to ensure that arrangements are in place to enable the timely transfer of TIPSS participants to the hubs to perform treatment. Prior to activating spokes, the REACT-AVB Trial Office will ensure that a regional hub has been activated.

#### Identification of patients

Patients will be identified by the clinical team through regular screening of patients admitted with variceal bleeding and emergency endoscopy procedures for gastrointestinal bleeding. Where patients are randomised in sites not offering TIPSS, those randomised to early TIPSS will be transferred to regional centres who do offer TIPSS and those randomised to standard of care will be managed as per routine practice, usually at the spoke site where they were first admitted.

For logistical reasons, outside of the site’s control, some participants who have been randomised to the TIPSS arm may not be able to receive the intervention (eg, due to unanticipated resource issues at site or an inability to conduct the procedure due to the patient’s health status) or may not receive the intervention within 4 days of diagnostic endoscopy. If this occurs, it will be documented on the follow-up case report form (CRF), and non-adherence will be monitored.

All participants will receive standard of care for initial control of bleeding consisting of resuscitation, endoscopic therapy within 24 hours of admission with variceal bleed, vasoactive drugs and antibiotics (figure 1).^3^

#### Consent process

Patients with capacity will be given sufficient time to read the patient information sheet (PIS) and ask questions and will be asked to sign the informed consent form (ICF), which will be countersigned by the PI. Where a patient has mental capacity but physically unable to sign the ICF, a witness will countersign the ICF.

There will be provisions for patients who lack capacity with support from another individual who will sign the ICF on behalf of the patient. The exact mechanism for this process will vary depending on the country of recruitment. The underlying principles of this process are that the individual is free to decide whether they wish to make the decision or not, considers the patient’s wishes setting aside own personal views and not doing so for remuneration or other personal gain. A separate information sheet, declaration form and ICF are provided for the individual and signed as appropriate with countersignature by the PI.

Where the patient regains capacity, consent will be sought from them by the research team at the earliest opportunity using recovered capacity PIS and recovered ICF. The patient’s wishes will supersede that of the individual who had previously consented on behalf of the patient when they lacked capacity.

Ongoing consent will be sought from the patient or the individual acting on behalf of the patient at each follow-up visit.

### Randomisation

Randomisation will be provided by Birmingham Clinical Trials Unit (BCTU) using a secure online system (available at: https://reactavb.bctu.bham.ac.uk/), thereby ensuring allocation concealment. Unique log-in usernames and passwords will be provided to those who wish to use the online system and who have been delegated the role of randomising patients into the trial as detailed on the REACT-AVB site signature and delegation log.

Participants with AVB will be randomised at the level of the individual, in a 1:1 ratio between early TIPSS and standard of care. A minimisation algorithm will be used within the online randomisation system to ensure balance in the intervention allocations over the following variables:

- Child-Pugh Score (7–9/10–13).
- Airway intubation for endoscopy (yes/no).
- Antibiotics administered (yes/no) (prior to or within 24 hours of diagnostic endoscopy).
- Terlipressin administered (yes/no) (prior to or within 24 hours of diagnostic endoscopy).
- Active bleeding seen at diagnostic endoscopy (yes/no).
- Recruiting centre.

To avoid the possibility of the intervention allocation becoming predictable, a random element will be included in the randomisation algorithm.

### Trial treatment/intervention

**Figure 2:**
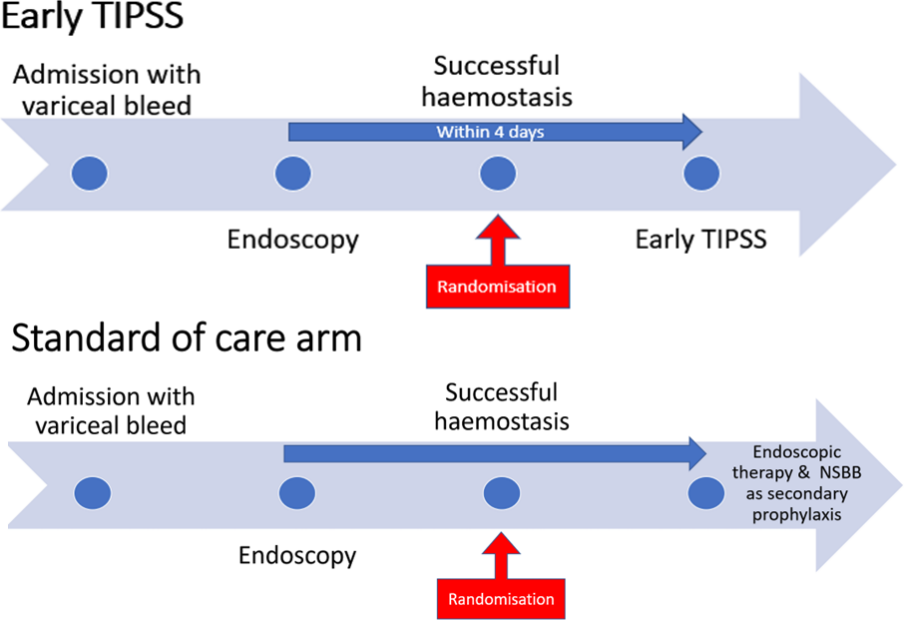
Details of health technologies. NSBB, non-selective beta blocker; TIPSS, transjugular intrahepatic portosystemic stent–shunt.

See trial treatment/intervention in figure 2.

#### Transjugular intrahepatic portosystemic stent–shunt

TIPSS is a procedure used in routine practice and should be undertaken in accordance with any applicable local policies and care pathways, ideally adhering to BSG guidance.^27^ The procedure is done under fluoroscopic guidance with the use of X-rays. Early TIPSS is delivered as per standard clinical practice and no trial specific mandates on administration apply. This includes any standard modifications for TIPSS occlusion or refractory hepatic encephalopathy. The time frame for early TIPSS is within 4 days of diagnostic endoscopy. It is the operator’s choice to decide whether general anaesthesia or deep sedation is most appropriate. Adherence to TIPSS will be defined as a participant receiving their TIPSS surgery within 4 days of diagnostic endoscopy. If TIPSS is not performed or is performed outside the specified time window, the reasons for this will be captured on a REACT-AVB CRF.

#### Standard care arm

Participants will undergo secondary prophylaxis against variceal rebleeding which usually comprises a combination of VBL and non-selective beta blocker (NSBB) as per the BSG guidelines.^3^ Modification (in line with local practice) may be needed due to intolerance of therapies, patient choice with regards to NSBB and banding or serious adverse event (SAEs).

Adherence to standard care will be defined as a participant not receiving early TIPSS within 10 days of diagnostic endoscopy for any indication. If participants in the standard of care arm do receive TIPSS, the reasons for this will be captured on a REACT-AVB CRF. The indications include rescue or salvage TIPSS, refractory or recurrent ascites and secondary prevention against variceal rebleeding.

Both treatments are currently in use in normal clinical practice and therefore the participant will continue to be treated after the trial has ended as per the standard care pathway.

### Outcome measures and study procedures

#### Primary outcome

Transplant-free survival at 1 year post randomisation.

#### Secondary outcomes

1. Transplant-free survival at 6 weeks (post randomisation).
2. Rebleeding (Rebleeding is defined as haematemesis and/or melena with either: (1) endoscopic evidence of variceal bleeding or stigmata of recent haemorrhage and at least a 2 g/L reduction in haemoglobin within 24 hours of admission or (2) massive upper gastrointestinal bleeding leading to death. The definition includes bleeding from banding ulceration.) (post randomisation):
  - Early (less than or equal to 6 weeks).
  - Late (greater than 6 weeks).
3. Serious adverse events related to treatment (up to 12 months post randomisation).
4. Other complications of cirrhosis (up to 12 months post randomisation; composite outcome and individual components):
  - New onset ascites.
  - New onset encephalopathy. (Hepatic encephalopathy will be assessed according to West Haven criteria as the most severe grade.^28^ The number of episodes of hepatic encephalopathy will be recorded in follow-up CRFs. Covert or minimal hepatic encephalopathy will be assessed using the animal naming test where less than 20 animals named in 1 min is highly suggestive of minimal or covert hepatic encephalopathy.^28^)
  - Spontaneous bacterial peritonitis.
  - Hepatocellular carcinoma.
  - Any renal dysfunction.
5. Child-Pugh Score at 6 and 12 months post randomisation.
6. MELD Score at 6 and 12 months post randomisation.
7. Cross-over therapies.
8. Health-related quality of life (EQ-5D-5L) at 6 and 12 months post randomisation.
9. Use of healthcare resources, costs and cost-effectiveness based on cost per quality-adjusted life-year (QALY) estimated using the EQ-5D-5L and cost per life year gained at 1 year and modelled cost per QALY over patient lifetime.

### Schedule of assessments

Schedule of assessments is detailed in table 1.

**Table 1:** Schedule of assessments

Assessment	Eligibility and randomisation	Baseline	2–7 days (TIPSS arm only)	6 weeks±7 days	6 months±7 days	12 months±7 days
Inclusion criteria*	x					
Exclusion criteria*	x					
Seek informed consent	x					
Relevant medical history taken	x					
Concomitant medication		x		x	x	x
Randomisation	x					
Full blood count		x		x	x	x
TIPSS procedure under fluoroscopic guidance†		x				
Renal function		x		x	x	x
Clotting profile	x			x	x	x
Liver function	x			x	x	x
Diagnostic endoscopy	x					
Standard clinical examination		x		x	x	x
Doppler ultrasound	x		x		x	x
Safety reporting		x	x	x	x	x
Quality of life questionnaires (EQ-5D-5L)		x			x	x
Child-Pugh Score ‡	x				x	x
MELD Score		x			x	x

Color significance HIghlight the assessment variable.

*Refer to text for inclusion and exclusion criteria.

†Early TIPSS arm only.

‡Child-Pugh Score is a minimisation variable.

EQ-5D-5L, EuroQol-5 Dimension-5 Level; MELD, Model For End-Stage Liver Disease; TIPSS, transjugular intrahepatic portosystemic stent–shunt.

### Statistical considerations

#### Sample size

The meta-analysis by Deltenre *et al* showed that early TIPSS results in an over 60% reduction in mortality compared with standard of care.^15^ However, we believe this is an overestimate when considering recent RCTs and propose a more conservative estimate.^10 14^ We have based our sample size on being able to detect a difference in transplant-free survival between two arms (standard care vs TIPSS) using a two-sided log-rank test. We have assumed a 1-year transplant-free survival rate in the standard care group of 60% and 80% in the TIPSS group (HR 0.44). To detect this difference with 90% power (alpha=0.05) and allowing for 20% attrition, we require 70 events across 294 participants (147 participants per arm).

#### Statistical analysis

A separate statistical analysis plan will provide a more comprehensive description of the planned statistical analyses. A brief outline of the planned analyses is given below.

The primary comparison groups will be composed of those randomised to standard of care versus those randomised to TIPSS. In the first instance, all analyses will be based on the intention-to-treat principle, that is, all participants will be analysed in the intervention group to which they were randomised irrespective of adherence to randomised intervention or protocol deviations. For all outcomes, appropriate summary statistics and differences between groups will be presented, with 95% CIs. Where possible, intervention effects will be adjusted for the minimisation variables as detailed earlier and baseline score (where appropriate). No adjustment for multiple comparisons will be made.

#### Primary outcome

The primary outcome will be compared between treatment groups using survival analysis methods. A Cox proportional hazard model will be fitted if the assumptions of proportionality are met, and an adjusted HR with a 95% CI will be presented. The p value relating to the intervention group parameter as generated by the model will be presented. Kaplan-Meier survival curves will be constructed for visual presentation.

#### Secondary outcome

Secondary outcomes which are considered time-to-event outcomes (transplant-free survival at 6 weeks) will be analysed in the same manner as the primary outcome (with the exception of p value reporting). Secondary outcomes which are considered binary (eg, rebleeding, complications of cirrhosis and use of cross-over therapies) will be summarised using frequencies and percentages. A log binomial regression model will be fitted and results presented as adjusted risk ratios, risk differences and 95% CIs. Continuous outcomes (eg, Child-Pugh and MELD scores) will be reported using means and SD at each time point. A mixed effects repeated measures model will be fitted and results presented as mean differences and 95% CIs at the primary time points (6 and 12 months). Longitudinal plots of the mean scores over time by treatment group will be produced for visual inspection of the data.

#### Planned subgroup analyses

Subgroup analyses will be limited to the same variables used in the minimisation algorithm as described above and performed on the primary outcome only. The effects of these subgroups will be examined by including an intervention group by subgroup interaction parameter in the regression model, which will be presented alongside the effect estimate and 95% CI within subgroups. The results of subgroup analyses will be treated with caution and will be used for the purposes of hypothesis generation only.

#### Missing data and sensitivity analyses

Every attempt will be made to collect full follow-up data on all participants; it is thus anticipated that missing data will be minimal. Any participants with missing primary outcome data up to 12 months will be included in the primary analysis up to the point where their clinical status (mortality and transplant data) is last known (censored at this point), therefore no sensitivity analyses to assess the impact of missing data are proposed for the primary outcome. Full details of any other sensitivity analyses will be included in the statistical analysis plan.

### Economic evaluation

The economic evaluation will assess the cost-effectiveness of early TIPSS versus standard of care (SOC) by calculating the cost per life year gained and cost per QALY gained over 12 months and lifetime from an NHS and Personal Social Services perspective.

A within-trial cost-effectiveness analysis will be conducted based on the outcomes of cost per life year gained and cost per QALY estimated using EQ-5D-5L responses at baseline, 6 and 12 months.^29^ Healthcare resource use information will be prospectively collected through CRFs and at regular follow-up hospital appointments. Resources include the intervention (early TIPSS) and standard of care (endoscopic treatment, medications), rebleeding, liver failure and transplantation, follow-up care including outpatient visits, investigations (eg, endoscopy), inpatient stays, admission to intensive care unit, treatment of adverse events and other complications and readmissions. Mean costs and outcomes will be estimated for both trial arms and non-parametric bootstrapping will be used to estimate 95% CIs around differences in mean costs, EQ-5D-5L scores and QALYs. In the base case, where there is missing cost and outcome data, multiple imputation will be used. Imbalances in baseline variables such as EQ-5D-5L Score between trial arms will be controlled for using a regression approach. Incremental cost-effectiveness ratios will then be calculated. Cost-effectiveness acceptability curves (CEACs) will be used to plot the probability of each intervention being cost-effective at different thresholds of willingness to pay per additional unit of outcome.

If there is evidence from the trial that differences between early TIPSS and SOC exist in terms of liver transplantation and mortality as well as other outcomes that may have significant cost or outcome implications beyond the trial period, a Markov model-based economic evaluation will also be conducted over a lifetime time horizon with discounting of 3.5% for costs and outcomes. Clinical and economic evidence collected as part of the trial and other secondary sources will be used to parametrise the model. Deterministic and probabilistic sensitivity analyses will be conducted to explore the robustness of the results and CEACs presented. Value of information analysis will also be undertaken to investigate the value of, and the need for, further information on key uncertain model parameters.

All methods and analyses will be reported as recommended by the Consolidated Health Economic Evaluation Reporting Standards reporting guidelines and full details of the analysis will be included in the health economics analysis plan.^30^

## Ethics and dissemination

### Ethical considerations

REACT-AVB was granted ethical approval by the research ethics committee (REC) of NHS (reference number: 23/WM/0085). This study involves human participants who gave informed consent to participate in the study before taking part. The trial will be conducted in accordance with the principles of Good Clinical Practice as defined by the UK Policy Framework for Health and Social Care Research and applicable UK Acts of Parliament and Statutory Instruments (and relevant subsequent amendments), which include Data Protection Act 2018 and Mental Capacity Act 2005. The protocol will be approved by the REC prior to the start of the trial. Before any patients are enrolled into the trial, the Principal Investigator (PI) at each site is required to obtain the necessary local approval.

### Publication plan

Outputs from this trial will be submitted for publication in peer-reviewed journals and the findings of the trial will be made public. Manuscripts will be prepared by the writing group as defined in the trial publication plan. Manuscripts should be submitted to the Trial Management Group in a timely fashion and in advance of being submitted for publication to allow time for review.

In all publications, authors should acknowledge that the trial was performed with the support of NIHR Health Technology Assessment and BCTU. Intellectual property rights will be addressed in the Clinical Study Site Agreement between sponsor and site.

## Data Availability

Data are available upon reasonable request. The final dataset will be available to members of the TMG and co-applicant group who need access to the data to undertake the final analyses. Requests for data generated during this study will be considered by BCTU. Data will typically be available 6 months after the primary publication unless it is not possible to share the data (eg, the trial results are to be used as part of a regulatory submission, the release of the data is subject to the approval of a third party who withholds their consent or BCTU is not the controller of the data). Only scientifically sound proposals from appropriately qualified research groups will be considered for data sharing. The request will be reviewed by the BCTU data sharing committee in discussion with the Chief Investigator and where appropriate (or in absence of the Chief Investigator) any of the following: the trial sponsor, the relevant Trial Management Group and independent TSC. A formal data sharing agreement (DSA) may be required between respective organisations once release of the data is approved and before data can be released. Data will be fully deidentified (anonymised) unless the DSA covers transfer of patient identifiable information. Any data transfer will use a secure and encrypted method.
